# Trigger warnings in medical student education: A scoping review

**DOI:** 10.1111/tct.13826

**Published:** 2024-11-04

**Authors:** James Fisher, Jessica Bennett, Abby Atkinson, Linda Errington

**Affiliations:** ^1^ School of Medicine Newcastle University Newcastle upon Tyne UK; ^2^ Northumbria Healthcare NHS Foundation Trust North Shields UK

## Abstract

**Background:**

Medicine is recognised as a challenging course where exposure to potentially distressing content is inevitable. Some educators provide students with warnings before they encounter potentially upsetting content—trigger warnings. In this scoping review, we mapped the existing literature seeking to better understand how trigger warnings are implemented in medical schools and how they are influencing education within them.

**Methods:**

Bibliographic databases were searched to identify relevant literature, including searching for grey literature. Articles were included if they focussed on medical school education and were written in English. Data analysis was undertaken using both quantitative and qualitative approaches (thematic analysis).

**Findings:**

Searching generated 1284 potential records for inclusion. Articles not related to the primary research question were excluded. Abstracts of the remaining 841 articles were screened, and ultimately, 12 articles met criteria for full‐text review. Of these, there were three empirical research studies. Qualitative analysis identified five main themes: purpose, implementation, student distress, responsibility and problems with terminology.

**Conclusions:**

The use of trigger warnings within medical student education remains contentious. There is a lack of consensus as to their purpose and much diversity in how they are implemented. There was limited published empirical evidence to inform practice in this area.

## 1 BACKGROUND

The process of studying medicine is recognised as being long, stressful and demanding for those embarking on a career in the field. Furthermore, the content covered within medical curricula can expose students to potentially traumatic events.^1^ These factors may go some way to explain why the prevalence of depression, anxiety and burnout amongst medical students is high.^2^, ^3^ Changes within medical schools over time are also of relevance. First, there is a recognition that the expansion in the numbers of medical students has led to some students feeling anonymous and isolated within their student cohort.^4^ Second, there has been a drive to widen participation in medical schools, with the aspiration that medical student populations become more representative of the demographic of the general population.^5^ Medical curricula have also evolved over time in an attempt to produce doctors who are more attuned to the needs of the populations they will ultimately serve. For example, topics such as racism, discrimination, inequality and abuse are recognised as important determinants of health with direct relevance to clinical encounters and thus now form part of medical curricula.^6^, ^7^ Sensitive topics such as these may have greater personal relevance to marginalised groups and thus have greater potential to cause discomfort.

Against this backdrop, the need for educators to be more mindful of student well‐being is clear, and many medical schools have implemented strategies to promote self‐care, reduce stress and provide social support for their students.^8^ There is also recognition that greater focus ought to be placed on the cultivation of a learning environment that is conducive to well‐being and mindful of students' differing prior experiences. Some have argued that educators should provide students with a warning before they encounter potentially upsetting content. Trigger warnings have been defined as ‘a statement preceding a piece of writing, video, etc. alerting the reader, viewer, etc., to the fact that it contains material or content that may cause distress, especially by reviving upsetting memories in people who have experienced trauma’.^9^ There is heterogeneity in how trauma is defined, but for the purposes of this work, we consider trauma to be: ‘any event that has had a lasting negative effect upon self and psyche’.^10^ The term ‘trigger’ is taken to mean something that causes distress in a vulnerable person and has its origins in psychology.^11^ The use of the term ‘trigger warnings’ began in feminist blog writing and in online forums as a way to alert readers that the material contained descriptions that might trigger, or exacerbate, post‐traumatic stress disorder and/or difficult emotions, in survivors of sexual abuse.^12^ Within the field of psychological science, a small empirical literature base on trigger warnings has emerged. A recent meta‐analysis demonstrated no effect on affective responses to negative material or on educational outcomes;^13^ however, these findings may not be generalisable to the field of medical education. The use of trigger warnings has since expanded beyond this original scope, and more recently, student calls for their use in higher education have brought trigger warnings to the fore. This has led to high‐profile debates about whether trigger warnings ought to have a role in university education.^11^, ^14^ Supporters argue that trigger warnings enable learners to prepare emotionally for distressing content, whereas critics contend that warnings enable learners to avoid challenging content and may even instil fear about said content.

In this scoping review, we set out to map the existing literature to better understand how trigger warnings are implemented in medical schools and how they are influencing education within them. Scoping reviews can be defined as ‘a type of knowledge synthesis (that) follow a systematic approach to map evidence on a topic and identify main concepts, theories, sources and knowledge gaps’.^15^ A scoping review is well‐suited to this topic area since the purpose of this work was to examine the range of literature published, identify knowledge gaps and clarify concepts. Whilst conducting this scoping review, we adopted an interpretivist epistemological standpoint. We acknowledged the absence of a single universal ‘truth’ in relation to trigger warnings and their use and instead sought to explore a breadth of viewpoints whilst recognising the influence of our own lived experiences in the process of data interpretation.

## 2 METHODS

A scoping review was undertaken in line with Arksey and O'Malley's five‐step approach: (i) identifying the research question; (ii) identifying relevant studies; (iii) study selection; (iv) charting the data; (v) collating, summarising and reporting the results.^16^ The Preferred Reporting Items for Systematic Reviews and Meta‐Analyses extension for scoping reviews (PRISM‐ScR)^15^ was employed to structure reporting of this scoping review.

i Identifying the research question

The population of interest in this review are medical students and their teachers. The situation of interest is the use of trigger warnings within the scholarly environment of medical schools. The research question was: *What is known about the impact of trigger warnings on learning in medical school education?*

ii Identifying the relevant studies

In March 2023, the following bibliographic databases were searched to identify relevant literature: British Education Index, Education Resources Information Centre (ERIC), Excerpta Medical Database (EMBASE), Ovid MEDLINE, PsycInfo, Scopus and Web of Science. The search strategy employed was developed in conjunction with a medical librarian and included keyword terms combined with Boolean operators: (1) trigger warning*.ti,ab,kf; (2) content warning*.ti,ab,kf; (3) content alert*.ti,ab,kf; (4) trigger alert*.ti,ab,kf; (5) or/1–4.

To access any ‘grey’ literature, the following bibliographic databases were also searched using the same search strategy: E‐Theses Online Service (EThOS), OpenGrey and ProQuest. The decision to include the grey literature, as well as all article types (e.g., opinion pieces, letters and commentaries), was constructively aligned with the exploratory nature of the review and the recognition that there was likely to be heterogeneity in views on trigger warnings. Articles were only included if they focussed on medical school education. No restriction on publication date was applied. Study inclusion and exclusion criteria are summarised in Table 1 below.

**TABLE 1:** Inclusion and exclusion criteria.

Criterion	Inclusion	Exclusion
Study design	All, including reviews, opinions, commentaries	
Population	Medical school education (students in a university context)	Education in other healthcare professions including medical education post‐university
Language	Published in English	Published in languages other than English
Source	Any, including grey literature	
Publication dates	All	

Reference lists and subsequent citations within all selected papers were also searched for any potentially relevant publications. The initial search was undertaken on 24 March 2023—repeat searches were undertaken, with the search process concluding 30 June 2023.

iii Study selection

**FIGURE 1:**
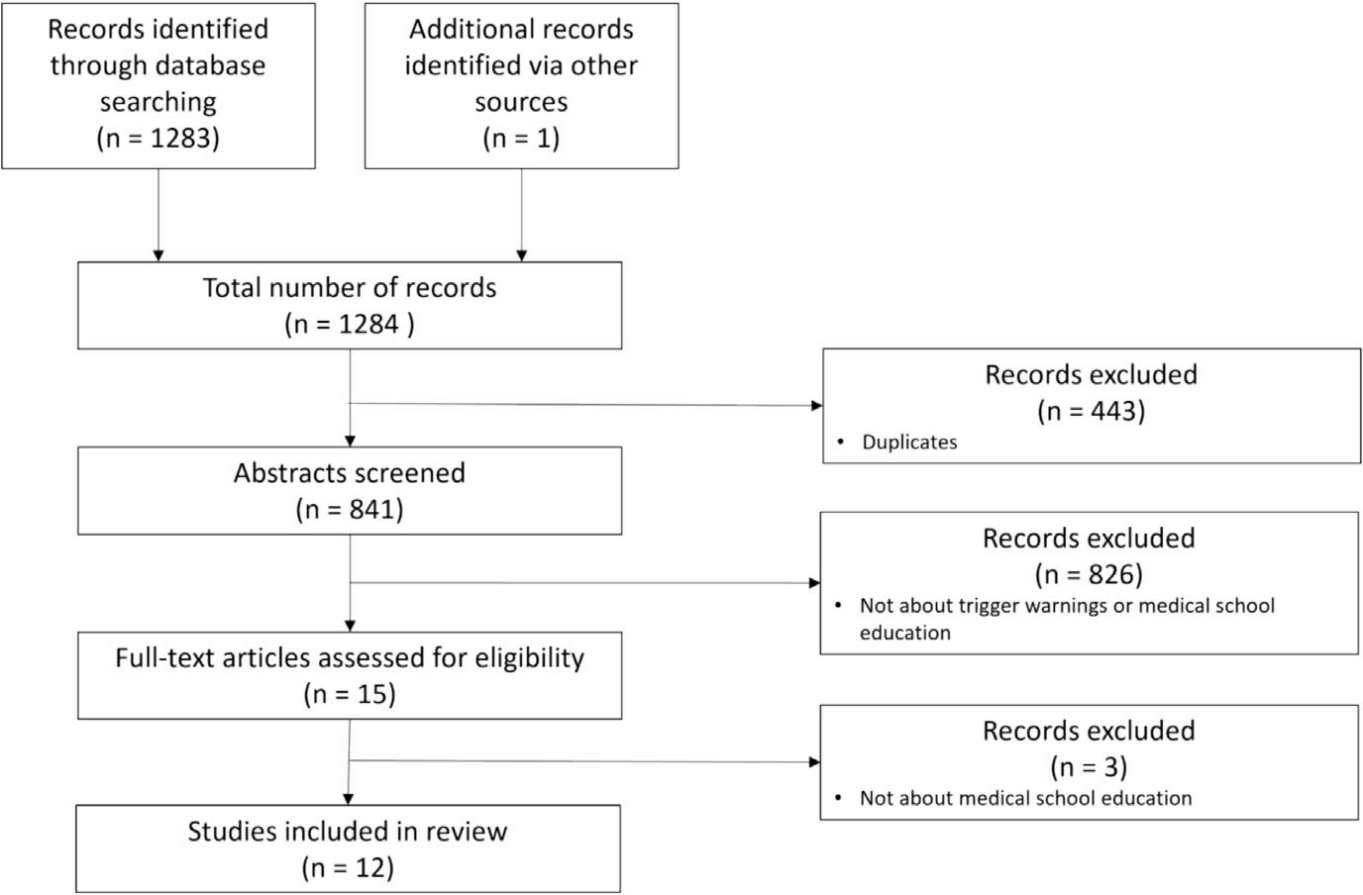
PRISMA flow diagram outlining search and selection processes.

Database searching generated a list of 1283 potential records for inclusion. One additional reference was identified from manual searching from reference lists. Data were merged using Rayyan, which facilitated identification and removal of duplicates. JB and AA both reviewed all abstracts of the remaining 841 articles to determine which were suitable for full‐text review, with JF acting as an adjudicator on any disagreements regarding eligibility. Articles written in languages other than English were excluded, since translation services were not available. Articles that were not related to the primary research question were also excluded at this stage. Fifteen articles met the criteria for full‐text review; however, on detailed review of these papers, a further three were excluded, as their focus was not aligned with the research questions posed by this scoping review. A flowchart outlining the search and selection processes, in line with PRISMA guidance, is displayed in Figure 1 below.

iv Charting the data

A spreadsheet was developed to allow charting of all eligible publications. In line with the process described by Thomas et al.,^17^ this process was iterative, with several cycles of developing, testing and revising needed before the final extraction form was agreed. JB and AA reviewed all articles and initially extracted data pertaining to authors' names, first author's location, year of publication, article type, stated aim and research questions, methodology employed, definitions of trigger warning and study findings. All such data were collated by two researchers with regular research meetings held to provide a forum for discussion and an opportunity to resolve any disagreements during the data charting process. Credibility of analysis was enhanced through prolonged engagement with the data and through investigator triangulation, with regular discussions enabling alternative interpretations of findings to be discussed, debated and agreed.

v Collating, summarising and reporting the results

Data analysis was undertaken using a mix of both quantitative and qualitative approaches. Basic quantitative and descriptive data, requiring low inference, were recorded for the following: authors' names, first author's location, year of publication, article type, stated aim and research questions, methodology employed and definitions of trigger warning. Qualitative analysis was undertaken using a thematic approach, as described by Braun and Clarke's framework.^18^ The six steps of this framework were employed as follows: (i) familiarising the data; (ii) generating initial codes; (iii) searching for themes; (iv) reviewing themes; (v) defining and naming themes; (vi) producing a report. To maintain coherence with the interpretivist approach employed in this work, researchers remained mindful that their views and experiences of trigger warnings were inextricably linked to their interpretation of the data. Our research team consisted of a medical sciences librarian and three medically trained clinical teachers, all of whom regularly deliver teaching to early‐stage medical students and therefore have lived experience of both teaching and learning at undergraduate level. Instead of seeking to ignore these experiences, researchers actively made them a topic for discussion within research group meetings in attempt to maintain a reflexive approach throughout.

## 3 FINDINGS

i Characteristics of included studies

Twelve articles were included in the scoping review. Publication dates, which we defined as the date when the work was first published online, ranged from 2016 to 2023, with the median year of publication being 2021. Over half of the articles (n = 7) were published between 2021 and 2023.^19^, ^20^, ^21^, ^22^, ^23^, ^24^, ^25^ In terms of geographical location, first authors were based in the United States (n = 6),^23^, ^24^, ^26^, ^27^, ^28^, ^29^ the United Kingdom (n = 5)^19^, ^20^, ^21^, ^22^, ^25^ and Canada (n = 1).^30^ Of the 12 articles, there were three empirical research studies—two qualitative case studies^20^, ^21^ and one cross‐sectional survey.^26^ Of the remaining articles, there were four opinion/commentary pieces,^24^, ^25^, ^29^, ^30^ three letters,^19^, ^22^, ^28^ one book section^27^ and one quality improvement initiative.^23^

The three empirical research studies recruited different participant populations. Nolan et al.^20^ purposively sampled educators who had current, or recent (defined as within 4 years), experience of delivering classroom‐based teaching to medical students in the early years of a graduate‐entry programme in the United Kingdom (n = 20). Nolan et al.^21^ latterly recruited students, drawn from years 1–3 of the same graduate‐entry medical degree programme in the United Kingdom (n = 13). Of note, participation was not limited to those students who identified as having had any previous traumatic experiences. Beverly et. al.^26^ invited 424 first and second year (preclinical) medical students on a US course to complete a survey, with a response rate of 61.1%. As with Nolan et al.,^21^ previous traumatic experience was not a prerequisite for participation.

Of the included articles, eight specified a definition of trigger warnings,^20^, ^21^, ^23^, ^24^, ^25^, ^26^, ^27^, ^30^ and four did not.^19^, ^22^, ^28^, ^29^ Neither of the two qualitative research papers^20^, ^21^ made reference to any theoretical framework to aid conceptualisation of trigger warnings and interpretation of their data nor was there any statement about the researchers' philosophical stance in relation to the phenomenon of interest.

Beverly et al.'s survey work^26^ provided quantitative data on students' perceptions of trigger warnings. Students were asked whether they had heard the term trigger warnings—only 11.2% of students reported having heard the term. When students were then provided with a definition and asked whether they had encountered them in their education, 38.6% reported having done so. There was no consensus as to whether trigger warnings ought to be used by medical schools (response/percentage: yes/31%, maybe/39.2%, no/29.7%). Students did not differ in their opinions on trigger warnings when their ethnicity, gender and stage of training were considered; however, students planning a career in family medicine were more likely to support their use.

ii Qualitative findings

**FIGURE 2:**
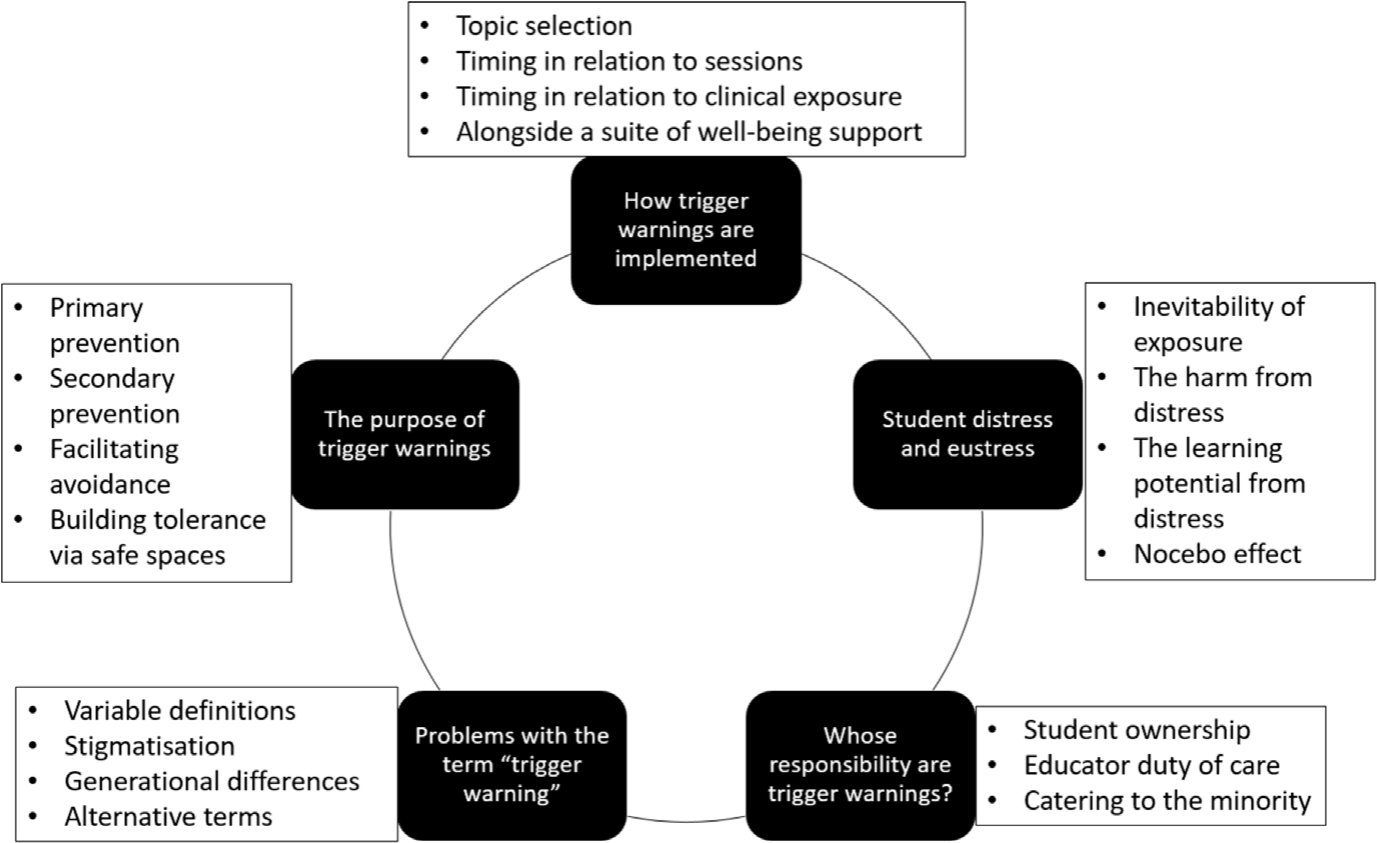
Overview of themes and subthemes.

Thematic analysis of the literature is presented using the five overarching categories that were identified during the analysis process—these are outlined, along with subthemes, in Figure 2 below.

### 3.1 The purpose of trigger warnings

There was diversity in the literature regarding the purpose of trigger warnings. Some authors' constructs of the purpose of trigger warnings aligned with their original purpose that of secondary prevention—to avoid re‐traumatisation for individuals with previous distressing experiences.^23^, ^24^ Such a definition was not universal though, with other authors considering trigger warnings to be a form of primary prevention—a method to avoid ‘educational iatrogenesis’, new harm caused as a result of the education process regardless of trauma history.^20^, ^21^, ^24^, ^25^, ^26^, ^27^, ^30^

Some raised concerns that the purpose of trigger warnings was to enable avoidance of any discomfort from discussion of topics that do not align with a student's world views.^20^, ^29^, ^30^ It was recognised that blanket avoidance of some topics in medical school would not be compatible with completion of one's studies.^20^ Others argued that trigger warnings facilitated attendance, since they enabled students to prepare psychologically for the upcoming session—in short, facilitating students to tolerate distressing topics, rather than avoid them.^20^, ^21^, ^28^

Authors highlighted how trigger warnings formed part of the education milieu in which students learnt by helping to create classrooms that are a ‘safe space’ for open exploration of challenging topics.^20^, ^23^, ^24^ In such environments, trigger warnings are recognised as being a signal to the student cohort that having an emotional response to course materials is acceptable and a valid topic for discussion.^20^, ^21^, ^23^, ^24^ Such warnings can prompt students to consider their emotional response to material and, crucially, empower students to seek support where appropriate.^20^, ^21^, ^28^, ^30^ Their use also challenges historic practices within medicine where self‐care is neglected, and a position of ‘detached concern’ is adopted.^20^ Some authors suggested their use may help nonaffected students develop a more nuanced understanding of trauma and thus be a stimulus for the development of empathy.^20^, ^21^, ^23^

### 3.2 How trigger warnings are implemented

There was a lack of consensus in the literature around what topic areas warranted a trigger warning. Topics where trigger warnings were advocated included: abuse,^27^ sexual violence,^25^, ^26^, ^27^, ^29^ domestic violence,^21^, ^27^ racism and racialised violence^21^; suicide,^20^, ^21^, ^27^ female reproductive health, for example, pregnancy loss, abortion, female genital mutilation,^23^, ^26^, ^27^, ^29^ death,^20^ addiction,^29^ eating disorders,^26^ gender,^20^ inequality,^20^ assault^29^ and severe injuries.^26^ The impossibility of being able to predict all potential triggers, given the individuality of students and their lived experiences, was highlighted.^20^ Arguments were made that widespread use of trigger warnings in view of this diversity might act to negate their utility as a result of students becoming desensitised to warnings and thus ignoring them.^20^

There was also variation in the timings of when trigger warnings were provided to students. Typically the approach was to provide warnings ahead of an upcoming session where topic areas were considered to be potentially traumatic.^19^, ^30^ Embedding discussion about trigger warnings within the process by which ground rules are established in small‐group teaching was also suggested.^30^ Alternatively, there was a description of an overarching warning provided within the course syllabus at the start of the academic year or within an induction period.^21^, ^23^ There was recognition that it is not possible to provide trigger warnings when learning in clinical settings due to their unpredictability^21^, ^26^, ^27^ and that their use early in the course may result in students less able to cope with distressing content encountered latterly in clinical practice.^26^ A graduated approach, whereby trigger warnings are gradually withdrawn as students' progress through the curriculum, was seen as a method to scaffold the development of students' self‐reliance and self‐awareness.^21^, ^26^

There was consideration of the impact of undergraduate and postgraduate status of medical students' need for trigger warnings. On one hand, it was contended that undergraduate, school‐leaver students had a greater need for them due to their relative lack of experience and maturity.^19^ However Nolan et al.,^21^ whose qualitative exploration of students' views on trigger warnings involved only graduate‐entry students, suggested that graduate‐entry students may have had more potentially ‘triggering’ life events by virtue of them being older.^22^ This study also acknowledges that older students, with more life experience, may have had more opportunities to develop coping strategies, compared to school‐leaver entrants. There was also a sense that trigger warnings ought not to be delivered in isolation—students feared trigger warnings might be employed instead of more meaningful emotional support.^21^ There was recognition amongst educators that student well‐being support was important and that there needed to be greater availability of such services, with trigger warnings forming part of a suite of resources.^19^, ^22^, ^23^, ^28^, ^29^

### 3.3 Student distress and eustress

There was widespread agreement in the literature that learning medicine meant that exposure to potentially distressing content was inevitable.^20^, ^21^, ^23^, ^26^, ^27^, ^29^ Whilst some students felt that choosing a career in medicine implied a readiness to deal with illness and suffering,^20^ there was recognition that medical school provided a period where students ought to learn to cope with distressing content before entering clinical practice.^20^, ^26^ Whilst there were fears that students, having received a trigger warning, might be sufficiently distressed that they then avoid potentially distressing material, there was no evidence of actual student avoidance within survey data from medical students^26^ nor from qualitative data from educators.^20^

Students described the distress caused by unexpectedly encountering material that reactivated memories of previous traumatic experiences.^26^ Non‐traumatised students were also able to empathise with such peers, imaging its potential impact on them.^21^ Discomfort with course content was recognised as being a fertile ground for learning, as such exposure to such content was a potentially a potent catalyst for self‐reflection and development.^20^, ^21^, ^30^ This ‘right amount’ of stress is sometimes termed eustress.^31^ Some authors considered the possibility that trigger warnings themselves might be a cause of harm, by hypersensitising students to content they had not previously considered to be traumatic—the so‐called nocebo effect.^20^, ^21^, ^25^

### 3.4 Whose responsibility are trigger warnings?

Within the literature, there was a range of views as to where the responsibility for student well‐being lay in respect to distressing course content. Some contended that by virtue of their status as medical students, it was the students' responsibility to accept potential exposure to distressing content and to put the needs of their patients before their own.^27^ Some students also felt that responsibility lay with them to disclose trauma at the outset of their studies, as opposed to waiting for warnings.^21^ Conversely, many felt that the duty of care for students lay primarily with educators and their institution.^20^, ^23^, ^24^, ^28^, ^30^ There was recognition that this duty of care had been brought into sharper focus by increasing diversity amongst the student body—the additional psychological burden amongst marginalised groups reinforced the need for educators to proactively take responsibility for their well‐being.^24^, ^30^ It was also evident that students have been proactively requesting trigger warnings.^20^, ^21^, ^25^, ^26^, ^28^, ^29^ Some contended that by acknowledging and agreeing to such a request, educators may be helping students develop their self‐awareness and encouraging the likelihood that they would seek support in challenging times.^20^, ^21^, ^28^, ^29^, ^30^ However, some students suggested that trigger warnings risked placing the focus of education on the minority (i.e., the small number of students who wanted trigger warnings),^21^ to the potential detriment of the wider student group through the introduction of bias in how such content was covered.^26^ There was also a concern that trigger warnings would be used defensively by educators to void themselves of responsibility for student response.^24^

### 3.5 Problems with the term ‘trigger warning’

There was a lack of consensus in relation to definitions for trigger warnings, as outlined in Table 2 below.

**TABLE 2:** Definitions for trigger warning contained within included papers.

Title	First author	Year	Definition
Cutting Close to the Bone: Student Trauma, Free Speech, and Institutional Responsibility in Medical Education	Kumagai, A.K.	2017	Notices ‐ verbal, written, or otherwise ‐ that instructors distribute to students before a specific discussion is held or reading assignment is given.
Students' Perceptions of Trigger Warnings in Medical Education	Beverly, E.A.	2018	Verbal statements or written warnings that alert students in advance to potentially distressing material.
Bioethics, Public Health, and the Social Sciences for the Medical Professions	Caruso Brown, A.E.	2019	Statements presented at the start of a piece of content alerting the user to the fact that it contains potentially distressing material.
Trigger Warnings in Medical Education	DeBonis, K.	2019	None
Several Way Generation Z May Shape the Medical School Landscape	Plochocki, J.H.	2019	None
Medical educators' views and experiences of trigger warnings in teaching sensitive content	Nolan, H.A.	2021	Offering prior notification of content so recipients may prepare for or avoid ensuing distress
On Triggering and Being Triggered: Civil Society and Building Brave Spaces in Medical Education	Wasserman, J.A.	2021	A warning about content that has the potential to be re‐traumatising to a person in light of specific events in their past (e.g., having been sexually assaulted).
Medical students' views on the value of trigger warnings in education: A qualitative study	Nolan, H.A.	2022	Prior notification allowing recipients to prepare for or avoid sensitive content and ensuing distress
Trauma‐Informed Care in the Classroom: Our Experience with a Content Warning in a Medical School Course	Stout, J.	2022	Warn learners of graphic and disturbing content that could cause intense physiological and psychological symptoms for people who experience post‐traumatic stress disorder and other anxiety disorders
When I use a word … Medical trigger warnings	Aronson, J.K.	2022	A statement cautioning that content (as in a text, video, or class) may be disturbing or upsetting.
Response triggered: Trigger warnings, a necessity or nuisance	Badawy, L.	2022	None
Response to Response Triggered: Trigger Warnings, a necessity or nuisance	Nolan, H.A.	2023	None

There was a recognition that the term ‘trigger warnings’ was associated with negative connotations. There was fear amongst students with current or previous mental health problems of being stigmatised as being weak and unable to cope.^21^ Some students considered the term to be disempowering,^21^ and educators also considered trigger warnings to be potentially infantilising.^20^ There was acknowledgement that generational differences were relevant to the use of trigger warnings. Both students and educators acknowledged than the current generation of students were more emotionally aware than previous generations,^20^, ^21^ with lower levels of psychological well‐being.^29^ Authors highlighted how debate around trigger warnings has sometimes descended into parody and distain for this generation, often as part of wider societal discussion about ‘political correctness’.^24^, ^29^, ^30^ Against this backdrop, and acknowledging some of the negativity surrounding use of the term, an argument was made to instead use the term ‘content warnings’.^20^

## 4 DISCUSSION

The aim of this scoping review was to explore the existing literature to better understand how trigger warnings are implemented in medical schools and how they are influencing education within them. The overarching finding from this work is that there is wide variation, and a lack of consensus, regarding the definition, purpose and implementation of trigger warnings within medical student education. This is compounded by a lack of empirical evidence in the context of medical education. Below, we outline the implications for medical education as well as sharing recommendations for educators, categorised by themes identified during analysis, in Table 3 below.

**TABLE 3:** Recommendations for educators organised by themes.

Theme	Recommendations for educators
Purpose	Challenge assumptions that trigger warnings drive avoidance amongst students
	Strive to reconceptualise trigger warnings as a tool to build tolerance of discomfort
Implementation	Seek agreement on what topics require trigger warnings through formalised discussion within staff‐student committees
	Seek consensus amongst educators on a consistent approach for timings and delivery of trigger warnings
	Ensure that support services are clearly signposted to students alongside trigger warnings
Distress	Be mindful of the Yerkes‐Dodson curve—aim for eustress, as opposed to distress
	Consider the diversity of student experience and how this might contribute to distress in the classroom setting
Responsibility	Proactively engage students with the development of ground rules within sessions
	To build empathy amongst students, harness the power of narrative medicine to craft learning experiences that give non‐traumatised students the opportunity to hear stories from individuals who have experienced trauma, particularly those from marginalised groups
Terminology	Seek to neutralise stigma associated with the term by making discussion around trauma a valid area for discussion
	Acknowledge that ageism towards younger generations is prevalent, particularly within the field of medicine

### 4.1 Implications for medical education

There was recognition that distress derails learning and can negatively impact on student well‐being. However, there was also acknowledgement that a degree of stress can provide a catalyst for learning—this ‘right amount’ of stress is sometimes termed eustress.^31^ Considering transformative learning theory, a theory that is recognised as being both relevant to and influential in medical education,^32^ may be instructive here. Transformative learning theory outlines how the discomfort brought by a ‘disorientating dilemma’^33^ can result in a change in viewpoint and thus learning. The Yerkes‐Dodson law—the inverted U‐shaped relationship between arousal and task performance^34^—is also relevant. This law suggests that optimal performance is achievable when arousal (stress) is at an intermediate level. If content is too disorientating or distressing, learning is stifled.

Evident within this review were perceptions and assumptions about younger generations. Such perceptions about the so‐called millennial and Gen‐Z generations are also seen within the media, where these generations are often pejoratively labelled as being self‐obsessed and lazy.^35^ The World Health Organisation's 2021 review on ageism^36^ highlighted how age is used to negatively categorise younger people, not just older people, and in Europe, those aged 15–24 years were the age group who reported experiencing the highest levels of ageism. Negative perceptions of younger people may be more common in medicine, where it is recognised that an engrained hierarchy of experience and seniority persists.^35^ We therefore call on educators to be mindful of ageism across generations and to reflect on how this might be influencing discussion and debate around the role of trigger warnings within medical education.

An important finding in this work was the absence of evidence to support the assertion that provision of trigger warnings led to students avoiding content entirely, although this finding comes with the caveat that there was limited empirical evidence uncovered by the scoping review. Recent empirical studies, albeit in a nonmedical setting, have eased concerns regarding avoidance of trigger‐warned material.^37^, ^38^ Interestingly, some have also suggested that trigger warnings might foster a ‘forbidden‐fruit effect’, whereby the warning increases, rather than decreases, attraction in such material.^13^ Our review reinforces the need for educators to consider trigger warnings as part of a wider strategic approach to maintaining student well‐being,^8^ as opposed to them being merely superficial statements. Proactive, formalised collaboration with students may help medical schools to reach consensus on the goal of trigger warnings, as well as determining which topics ought to be covered.

Being explicit with students about the purpose of trigger warnings and seeking to nurture a collaborative approach with students through co‐development of ground rules may help to conceptualise trigger warnings as a tool to build tolerance of discomfort, as opposed to barriers to learning. Narrative medicine may have a role to play in helping non‐traumatised students develop a deeper understanding of the impact of trauma. Rita Charon, originator of the field of narrative medicine, termed it ‘a commitment to understanding (patients') lives … giving voice to the suffering’.^39^ In essence, teaching using narrative medicine can ‘reveal worlds that are otherwise closed to us’.^40^ Harnessing this may help build empathy amongst students for their peers, but also for the diverse populations they will ultimately serve as doctors.^41^

### 4.2 Directions for future research

This scoping review identified limited empirical evidence for the use of trigger warnings within medical student education. Future research should explore the lived experiences of undergraduate medical students when encountering potentially traumatic content in their learning, since there was a lack of their voice within the literature. Co‐production initiatives, where students are meaningfully engaged as cocreators of curricula or research, may provide a fruitful approach.^42^ Such research may also be enhanced through adoption of a systems‐based approach, where there is engagement of key stakeholders across campus and clinical settings, consideration of the breadth of the curriculum and the environment in which it is delivered, and acknowledgment of the diversity of both students and staff and its impact on potentially traumatic material.

### 4.3 Review limitations

There are some limitations to this review. First, the review did not explicitly assess quality of the included studies, although some relevant methodological limitations were acknowledged. Whilst this is in line with accepted scoping review methodology,^43^ this ought to be kept in mind when readers seek to extrapolate findings from the work. Second, despite employing a comprehensive search of bibliographic databases, it is possible that some relevant articles may have been missed.

## 5 CONCLUSION

This scoping review has demonstrated that the use of trigger warnings within medical student education is extremely variable. There is a lack of consensus in the literature in relation to the purpose of trigger warnings and about what topics necessitated the use of trigger warnings. Furthermore, there was diversity in how trigger warnings were implemented into medical student education. There is limited published empirical evidence to inform practice in this area, and thus, further research is required to better understand how trigger warnings can be integrated into the experiences and development of medical students.

## AUTHOR CONTRIBUTIONS

Jessica Bennett, Abby Atkinson and Linda Errington developed the search strategy. Jessica Bennett and Abby Atkinson completed the literature search and independently reviewed titles and abstracts of all articles. James Fisher prepared the initial manuscript draft. All authors provided input into the drafting process. All authors read and approved the final manuscript.

## CONFLICT OF INTEREST STATEMENT

The authors declare that they have no competing interests.

## ETHICS STATEMENT

Ethical approval was not required for this article given that it is a review of previously published literature and does not involve any human subjects.

## Data Availability

All data generated or analysed during this study are included in this published article.
